# The Short-Term Effect of Weight Loss Surgery on Volumetric Breast Density and Fibroglandular Volume

**DOI:** 10.1007/s11695-016-2415-6

**Published:** 2016-10-26

**Authors:** Nasreen A. Vohra, Swapnil D. Kachare, Paul Vos, Bruce F. Schroeder, Olga Schuth, Dylan Suttle, Timothy L. Fitzgerald, Jan H. Wong, Kathryn M. Verbanac

**Affiliations:** 1 Department of Surgery, Brody School of Medicine, East Carolina University, Greenville, NC 27834 USA; 2 Vidant Medical Center, Greenville, NC 27834 USA; 3 Department of Biostatistics, College of Allied Health Sciences, East Carolina University, Health Sciences Building, Greenville, NC 27834 USA; 4 Carolina Breast Imaging Specialists, 990, Johns Hopkins Dr, Greenville, NC 27834 USA; 5 Department of Surgery, Virginia Commonwealth University School of Medicine, Richmond, VA USA; 6 Department of Radiology and Medical Imaging, University of Virginia, Charlottesville, VA USA

**Keywords:** Weight loss surgery, Fibroglandular volume, Volumetric breast density, Breast density, Breast cancer, Obesity, Diabetes, Breast Volume

## Abstract

**Purpose:**

Obesity and breast density are both associated with an increased risk of breast cancer and are potentially modifiable. Weight loss surgery (WLS) causes a significant reduction in the amount of body fat and a decrease in breast cancer risk. The effect of WLS on breast density and its components has not been documented. Here, we analyze the impact of WLS on volumetric breast density (VBD) and on each of its components (fibroglandular volume and breast volume) by using three-dimensional methods.

**Materials and Methods:**

Fibroglandular volume, breast volume, and their ratio, the VBD, were calculated from mammograms before and after WLS by using Volpara™ automated software.

**Results:**

For the 80 women included, average body mass index decreased from 46.0 ± 7.22 to 33.7 ± 7.06 kg/m^2^. Mammograms were performed on average 11.6 ± 9.4 months before and 10.1 ± 7 months after WLS. There was a significant reduction in average breast volume (39.4 % decrease) and average fibroglandular volume (15.5 % decrease), and thus, the average VBD increased from 5.15 to 7.87 % (*p* < 1 × 10^−9^) after WLS. When stratified by menopausal status and diabetic status, VBD increased significantly in all groups but only perimenopausal and postmenopausal women and non-diabetics experienced a significant reduction in fibroglandular volume.

**Conclusions:**

Breast volume and fibroglandular volume decreased, and VBD increased following WLS, with the most significant change observed in postmenopausal women and non-diabetics. Further studies are warranted to determine how physical and biological alterations in breast density components after WLS may impact breast cancer risk.

## Introduction

Mammographic density is a term used to describe the proportion of radiopaque, fibroglandular/dense tissue on a mammogram. Over the last few decades, there has been increasing attention to the association between mammographic density and the risk of breast cancer development. In 1976, Wolfe first reported that breast cancer risk was associated with mammographic parenchymal patterns [1]. Although initially this increased risk was attributed to a “masking bias” stemming from difficulty in detecting a tumor against a radiodense background, further studies confirmed that a higher density in and of itself conferred additional risk [2]. Multiple studies have consistently shown a twofold to sixfold increased risk of breast cancer in women with the highest measures of mammographic density compared to the lowest [3, 4]. It is becoming evident that density is a dynamic phenotype of processes occurring in the body and is influenced by many factors including age, menopausal status, parity, hormonal changes, body mass index (BMI), and metabolic changes [5–8]. Several studies have now shown that reductions in mammographic density over time, for example, in response to endocrine therapies, are associated with a significant decrease in breast cancer risk [9–15]. Because density is a modifiable risk factor and can be targeted for cancer risk reduction, there is a great deal of interest in investigating the biologic and molecular mechanisms that link breast density to breast cancer risk.

The increased absolute risk of postmenopausal breast cancer with increasing BMI is well known, although the reason for this association is not clear [16]. Large epidemiologic studies have shown that weight loss surgeries (WLS), like Roux-en-Y gastric bypass, reduce the risk of postmenopausal breast cancer [17–20]. Weight loss surgery not only results in a drastic reduction in BMI but also often reverses diabetes and many components of the metabolic syndrome [21, 22]. Metabolic syndrome and insulin resistance are associated with mammographic dense breasts [23]. In most patients, WLS causes a significant reduction in body fat, which is expected to increase breast density due to the inverse relationship of mammographic density with BMI [24]. Yet, paradoxically, a decrease in breast cancer risk in fact occurs after WLS. There have been no published studies reporting the impact of WLS on breast density and whether both components that comprise density, i.e., the fibroglandular tissue and the adipose tissue, are affected. WLS is likely to change both these components that make up density, due to changes in growth factors and hormones such as insulin-like growth factor-1 (IGF-1), and estrogen levels after surgery [25–27]. With improvements in metabolic regulation after WLS, changes in the fibroglandular compartment of the breast may also significantly contribute to the reduction in breast cancer risk.

Clinically, the most widely used method for assessing mammographic density is the Breast Imaging Reporting and Data System (BI-RADS) composition categories, a subjective measure of the extent to which the amount of dense tissue in the breast could potentially obscure small lesions [28]. BI-RADS density categories are fairly broad (∼80 % of women fall into the middle two categories) and may not be sensitive enough to detect clinically meaningful changes in mammographic density [29]. Most of the recent epidemiological studies investigating mammographic density and breast cancer risk have chosen to use quantitative visual assessments or semi-automated or fully automated methods for quantifying breast density. However, one study looking at mammographic density reduction after tamoxifen by Cuzick et al. noted that the minimum change that could reproducibly be detected by using a quantitative visual mammographic density scale was 10 % [10].

Recent advances in quantifying mammographic density by using volumetric methods have several advantages over visual and two-dimensional area-based measures. Volumetric methods capture information that represents the three-dimensional nature of breast tissue. Highly correlated to ground truth measurements from MRI, volumetric methods show good reliability across serial mammograms and are significant predictors of breast cancer risk [30–36]. Commercial volumetric methods can also output a volumetric density grade analogous to the BI-RADS categories and have been validated compared to visual assessment [37–40].

In the present single-institution retrospective study, using volumetric density measures, we analyzed the impact of WLS on breast density and on each of the components that determine breast density.

## Methods

This study is a retrospective analysis to determine the change in breast density measured from full-field digital mammography images by using the Volpara method (Algorithm version 1.4, Volpara Solutions, Wellington, New Zealand) in women undergoing WLS. The study was approved by the Institutional Review Board at East Carolina University and was in compliance with the Health Information Portability and Accountability Act.

### Subjects

**Fig. 1:**
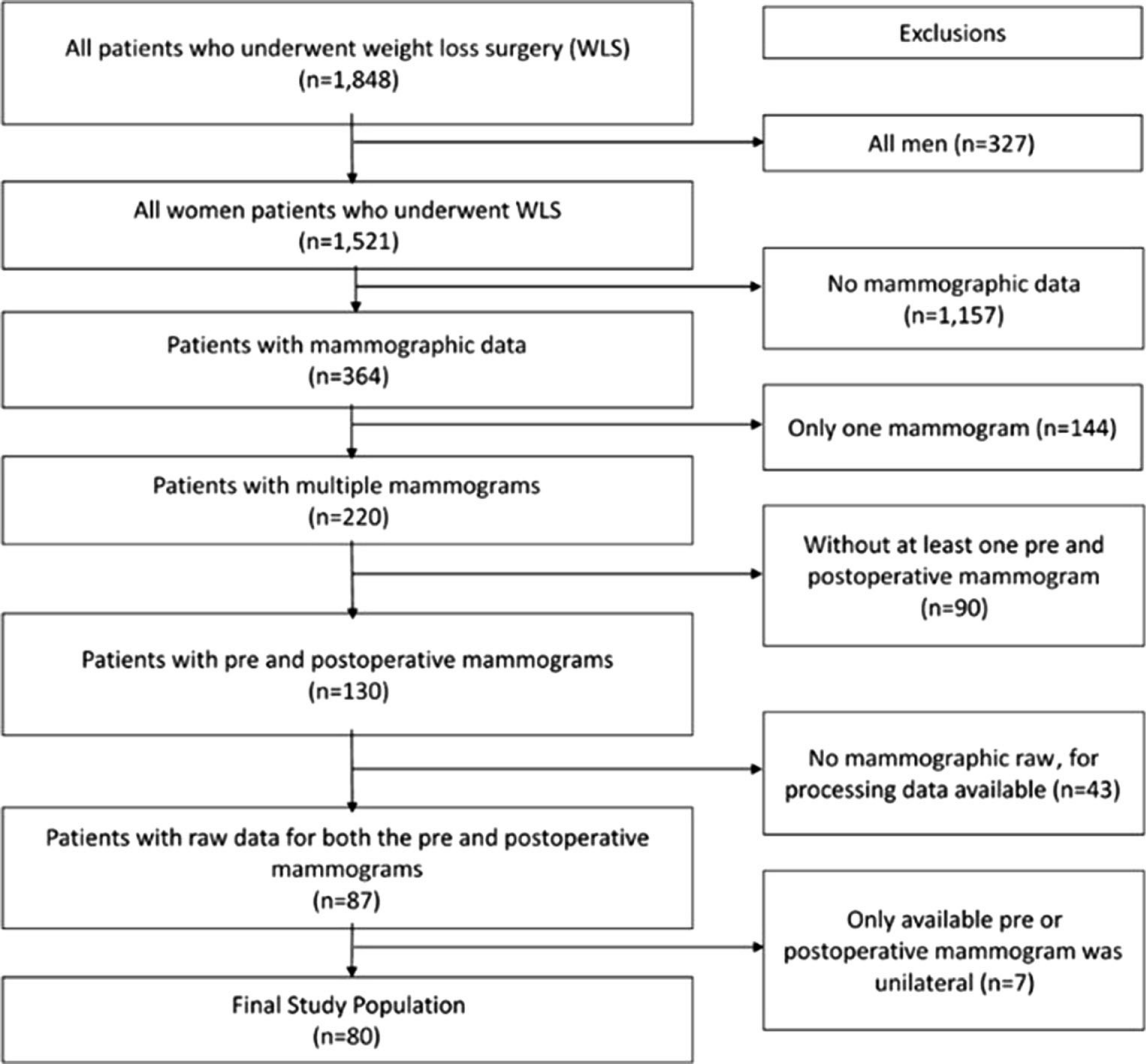
Study population

All patients who underwent WLS from 2003 to 2013 at Vidant Medical Center by bariatric surgeons at The Brody School of Medicine, East Carolina University in Greenville, North Carolina, were identified. As shown in Fig. 1, a total of 1848 patients either underwent a gastric bypass, gastric banding, or sleeve gastrectomy. All men (*n* = 327), patients without any mammographic data (*n* = 1157), patients with only one mammogram (*n* = 144), and patients without both a preoperative and postoperative mammogram (*n* = 90) were excluded. Of the 130 patients who met the inclusion criteria, 43 patients were excluded because raw processing mammographic data were not available. An additional seven patients were excluded because the only preoperative or postoperative mammogram available was a unilateral mammogram. Eighty patients had the necessary radiologic data to be included in the study.

### Assessment of Volumetric Breast Density Using Volpara™

Volpara™, previously described by Aitken et al., is an FDA-cleared, fully automated software used for estimating volumetric breast density [41, 42]. Volpara™ analyzes digital mammographic data in a volumetric fashion and produces a quantitative assessment of breast composition by using fibroglandular volume (FGV), total breast volume, and their ratio to determine volumetric breast density (VBD). Pre-WLS and post-WLS mammograms were identified for each patient. When multiple presurgery mammograms were available, the images captured closest to the surgery date were used for density measurements. For the postoperative mammogram, the first mammogram that was acquired at least 60 days after surgery was used. Raw mammographic data for craniocaudal and mediolateral oblique views of preoperative and postoperative bilateral screening mammograms performed at a single breast imaging center were obtained to calculate average compressed breast thickness, total breast volume, FGV, and VBD by using the Volpara imaging software. Using preset thresholds of VBD, Volpara also outputs a Volpara density grade (VDG) corresponding to the BI-RADS density categories (i.e., VDG 1 VBD <4.5 %, VDG 2 VBD ≥4.5 and <7.5 %, VDG 3 VBD ≥7.5 and ≤15.5 %, VDG 4 VBD >15.5 %) which are also reported in the analysis.

For women who did not have menopausal status clearly documented in the medical record (*n* = 38), age <45 years was considered premenopausal, between 45 and 55 years as perimenopausal, and >55 years as postmenopausal. Women who changed menopausal status during the study period were included in the perimenopausal group.

### Statistical Analysis

Changes in breast density and other numeric variables were analyzed by using the one-sample *t* test with 95 % confidence intervals for the mean change. Box plots revealed a few moderate outliers for some of the variables, but we found no indications of any gross violations of the analyses based on the *t* test. In some cases, the tests were repeated on the log scale with no changes in statistical significance at the 5 % level. The postsurgery measurements were taken between 2 and 40 months after surgery; however, scatter plots of the changes plotted against the time of measurement did not show an important trend. These analyses were repeated on subgroups defined by menopausal status, diabetes status, and race. Change in VBD for patients having both pre-WLS and post-WLS BI-RADS density scores of 2 was also independently analyzed. Analyses were performed by using RStudio (version 0.98.501, Boston, MA, http://www.rstudio.com) and R (version 3.0.2 with the mosaic package, http://www.R-project.org/).

## Results

### Characteristics of the Study Population

As shown in Fig. 1, 80 patients had the necessary radiologic data to be included in the study. Table 1 highlights the characteristics of the study population, and Table 2 compares relevant patient variables at the time of their pre-WLS and post-WLS mammogram. The mean BMI at the time of the preoperative mammogram was 46.0 ± 7.22 kg/m^2^; this decreased to 33.7 ± 7.1 kg/m^2^ at the time of the postoperative mammogram. The most common WLS was a laparoscopic gastric bypass with 62 (78 %) patients undergoing it. The other types of WLS performed included open gastric bypass, laparoscopic sleeve gastrectomy, and gastric banding. Through the study period, 16 (20 %) women were premenopausal, 27 (33.8 %) were perimenopausal, and 37 (46.2 %) were postmenopausal. The preoperative mammogram was performed on an average of 11.6 ± 9.4 months prior to weight loss surgery. On average, the postoperative mammogram was performed 10.1 ± 7.0 months post-WLS; this time interval ranged from 2 to 40 months. Thirty-five (44 %) patients had type II diabetes mellitus prior to surgery. Resolution of diabetes was observed in 21 (60 %) of these patients after surgery.

**Table 1:** Characteristics of the women in the study

Variable	*N* (%)
Race	
White	44 (55)
African American	36 (45)
History of contraceptive use	
No	69 (86.3)
Yes	11 (13.8)
Hormone replacement therapy	
No	68 (85.0)
Yes	12 (15.0)
Family history of breast cancer	
No	41 (51.3)
Yes	39 (48.8)
Alcohol use	
No	72 (90.0)
Yes	3 (3.8)
Unknown	5 (6.2)
Smoking history	
Never	51 (63.8)
Former	23 (28.7)
Active	6 (7.5)
Weight loss surgery	
Lap gastric bypass	62 (77.5)
Lap sleeve gastrectomy	8 (10.0)
Lap gastric band	7 (8.8)
Open gastric bypass	3 (3.7)

**Table 2:** A comparison of characteristics of the study population at the time of the pre-weight and post-weight loss surgery mammograms

Variable	Pre-WLS mammogram *N* (%)	Post-WLS mammogram *N* (%)	*p* value
Total	80	80	
Age (years)			
Mean ± SD	51.4 ± 7.70	53.2 ± 7.46	0.13
Median (range)	51.4 (35.8–67.0)	53.4 (38.2–68.4)	
BMI (kg/m^2^)			
Mean ± SD	46.0 ± 7.22	33.7 ± 7.06	<0.0001
Median (range)	44.4 (35.1–68.2)	33.5 (18.5–54.3)	
Diabetes			
Yes	35 (43.8)	14 (17.5)	0.002
No	45 (56.2)	66 (82.5)	
Insulin use			
Yes	10 (12.5)	4 (5.0)	0.09
No	70 (87.5)	76 (95.0)	
BI-RADS score^a^			
0	2 (2.8)	1 (1.3)	0.62
1	39 (54.9)	41 (53.2)	
2	30 (42.3)	34 (44.2)	
4c	0	1 (1.3)	
BI-RADS density^a^			
1 Almost entirely fat	4 (5.6)	7 (9.1)	0.43
2 Scattered fibroglandular densities	52 (73.2)	49 (63.6)	
3 Heterogeneously dense	15 (21.1)	20 (26.0)	
4 Extremely dense	0	1 (1.3)	
Type of mammogram			
Screening	59 (73.8)	70 (87.5)	0.002
Diagnostic	13 (16.2)	10 (12.5)	
Unknown	8 (10.0)	0	

^a^Information not available on all participants

### Total Breast Volume and Compressed Breast Thickness Decrease After Weight Loss Surgery

In the entire study population, the total breast volume decreased an average of 579.7 ± 444 cm^3^ from 1469.89 to 890.17 cm^3^ (a significant 39.4 % decrease, *p* < 2.2e-16). Both adipose volume and FGV make up the total breast volume. This extreme reduction in the total breast volume is consistent with the significant weight loss and decrease in BMI in these patients. The average decrease in compressed breast thickness was 22.36 ± 12.67 mm (*p* < 2.2e-16) from 67.56 to 45.20 mm. This trend in reduction of total breast volume and compressed breast thickness was observed in women regardless of menopausal status.

### Fibroglandular Volume Decreases After Weight Loss Surgery

**Fig. 2:**
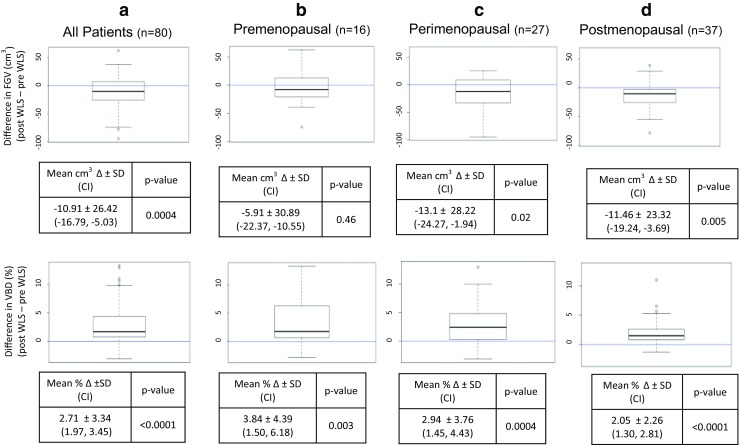
Difference in fibroglandular volume (FGV, *top panel*) and volumetric breast density (VBD, *bottom panel*) before and after weight loss surgery in the **a** entire study population, **b** premenopausal women, **c** perimenopausal women, and **d** postmenopausal women. *N* number of patients in each group. *Bold line* within the box plot indicates median

The mean FGV on the preoperative and postoperative mammograms was 70.5 ± 30.6 and 59.6 ± 27.64 cm^3^, respectively, with an average decrease of 15.5 % (*p* = 0.0004, 95 % CI −16.78, −5.03) as shown in Fig. 2, top panel a. When stratified by menopausal status (Fig. 2, top panel b, c, d), the premenopausal group had an average decrease in FGV of 5.9 ± 30.9 cm^3^, but this change was not statistically significant (*p* = 0.46). However, both the larger perimenopausal (*n* = 27) and postmenopausal (*n* = 37) cohorts had a greater and significant decrease in FGV of 13.1 ± 28.2 cm^3^ (*p* = 0.02) and 11.5 ± 23.3 cm^3^ (*p* = 0.005), respectively. The average times to mammogram for the perimenopausal and postmenopausal groups were 11 ± 8.1 and 9 ± 6.5 months after WLS, respectively. Adipose volume was calculated as the difference in the total breast volume and FGV. As expected after WLS, adipose volume decreased significantly in all groups (data not shown). The mean adipose volume for the entire population before and after surgery was 1399.41 ± 577.7 and 830.6 ± 475.17 cm^3^, with the mean decrease in the total adipose volume being 568.82 ± 431.19 cm^3^ (*p* < 1 × 10^−15^).

### Volumetric Breast Density Increases After Weight Loss Surgery Regardless of Menopausal Status

For the entire study population, the mean percent VBD prior to undergoing WLS was 5.2 ± 2.04 % (range, 2.1 to 13.4 %) and increased to 7.9 ± 3.90 % (range, 2.6 to 21.1 %) after WLS (*p* < 0.0001). The increase in VBD was significant regardless of the menopausal status (Fig. 2, lower panel).

In the 16 patients who were premenopausal prior to surgery and remained premenopausal at the time of follow-up mammogram, average breast density increased by 3.8 %, from 4.9 to 8.7 % (*p* = 0.003). Twenty-seven patients in the perimenopausal group also had an increase in breast density with an average increase in density from 5.3 ± 1.69 to 8.20 ± 4.20 % (*p* = 0.0004). The 37 individuals who were postmenopausal prior to WLS also had a significant increase in breast density from 5.2 to 7.2 % (*p* < 0.0001).

### Volumetric Breast Density Increases Regardless of Diabetic Status, but only Non-Diabetics Experience a Significant Decrease in Fibroglandular Volume

**Fig. 3:**
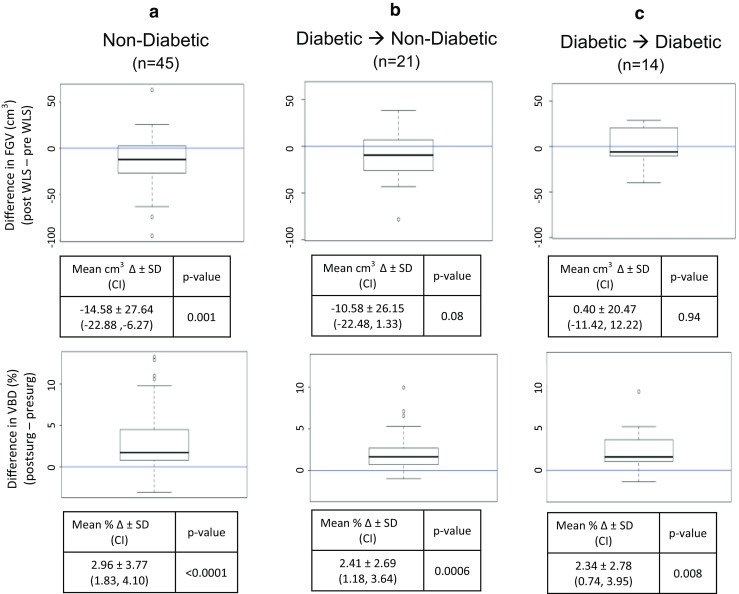
Difference in fibroglandular volume (FGV, *top panel*) and volumetric breast density (VBD, *bottom panel*) before and after weight loss surgery in **a** non-diabetics, **b** diabetics who became non-diabetics, and **c** diabetics who stayed diabetic. *N* number of patients in each group. *Bold line* within the box plot indicates median

Prior to the operation, 35 patients (44 %) had type 2 diabetes mellitus, nine of whom were on insulin. At the time of their postoperative mammogram, 14 (18 %) had diabetes, with only 4 taking insulin. Twenty-one patients (60 % of diabetics) had resolution of their diabetes postoperatively. Regardless of the diabetic status, there was a significant increase in breast density as shown in Fig. 3, lower panel. As shown in Fig. 3, upper panel, non-diabetics (*n* = 45) prior to surgery had a significant mean reduction in FGV of 14.6 cm^3^ (*p* = 0.001). Similarly, 21 patients who had resolution of their diabetes also had a reduction in FGV of 10.6 cm^3^ (*p* = 0.08). In contrast, the FGV of those patients who remained diabetic at the time of their postoperative mammogram was not significantly different (0.4-cm^3^ mean increase; *p* = 0.94). There was no significant difference in the timing of the postoperative mammograms; non-diabetics underwent a postoperative mammogram on average 9.5 ± 6.4 months from surgery compared to the diabetic group who underwent mammogram 10.9 ± 7.8 months after surgery (*p* = 0.42).

### Volumetric Breast Density Increased and Fibroglandular Volume Decreased Regardless of Race

Both white and African American women, who were well represented in the study, had a significant increase in breast density after WLS (*p* < 0.0001 for the entire study population). African American women had a 13.3-cm^3^ reduction in FGV (*p* = 0.005), and white women had a 9.0-cm^3^ reduction in FGV (*p* = 0.03). A statistically significant difference was not observed in the amount of reduction in FGV between African American and white women.

### Change in BI-RADS Density Score After Weight Loss Surgery

We analyzed change in BI-RADS density pre and post WLS. The BI-RADS 2 (scattered fibroglandular density) density category was the most common both preoperatively and postoperatively (52 and 49 patients, respectively), followed by BI-RADS 3 (heterogeneously dense; 15 and 20 patients, respectively) (Table 3). Nine patients did not have a BI-RADS density score available for the preoperative and/or the postoperative mammogram. There was no important change evident in BI-RADS density scores after WLS; pre-WLS and post-WLS BI-RADS scores remained the same for 45 of the 80 patients, increased by 1 in 12 patients, increased by 2 in 1 patient, and decreased by 1 in 13 patients.

**Table 3:** BI-RADS density score pre and post weight loss surgery

		Post					
		BI-RADS 1	BI-RADS 2	BI-RADS 3	BI-RADS 4	Unknown	Total
Pre	BI-RADS 1	**0**	4	0	0	0	4
	BI-RADS 2	7	**36**	8	1	0	52
	BI-RADS 3	0	6	**9**	0	0	15
	BI-RADS 4	0	0	0	**0**	0	0
	Unknown	0	3	3	0	3	*9*
	Total	7	49	20	1	3	80

Bold boxes indicate the number of patients with the same BI-RADS density scores pre and post WLS

### BI-RADS Density Versus Volumetric Breast Density

**Fig. 4:**
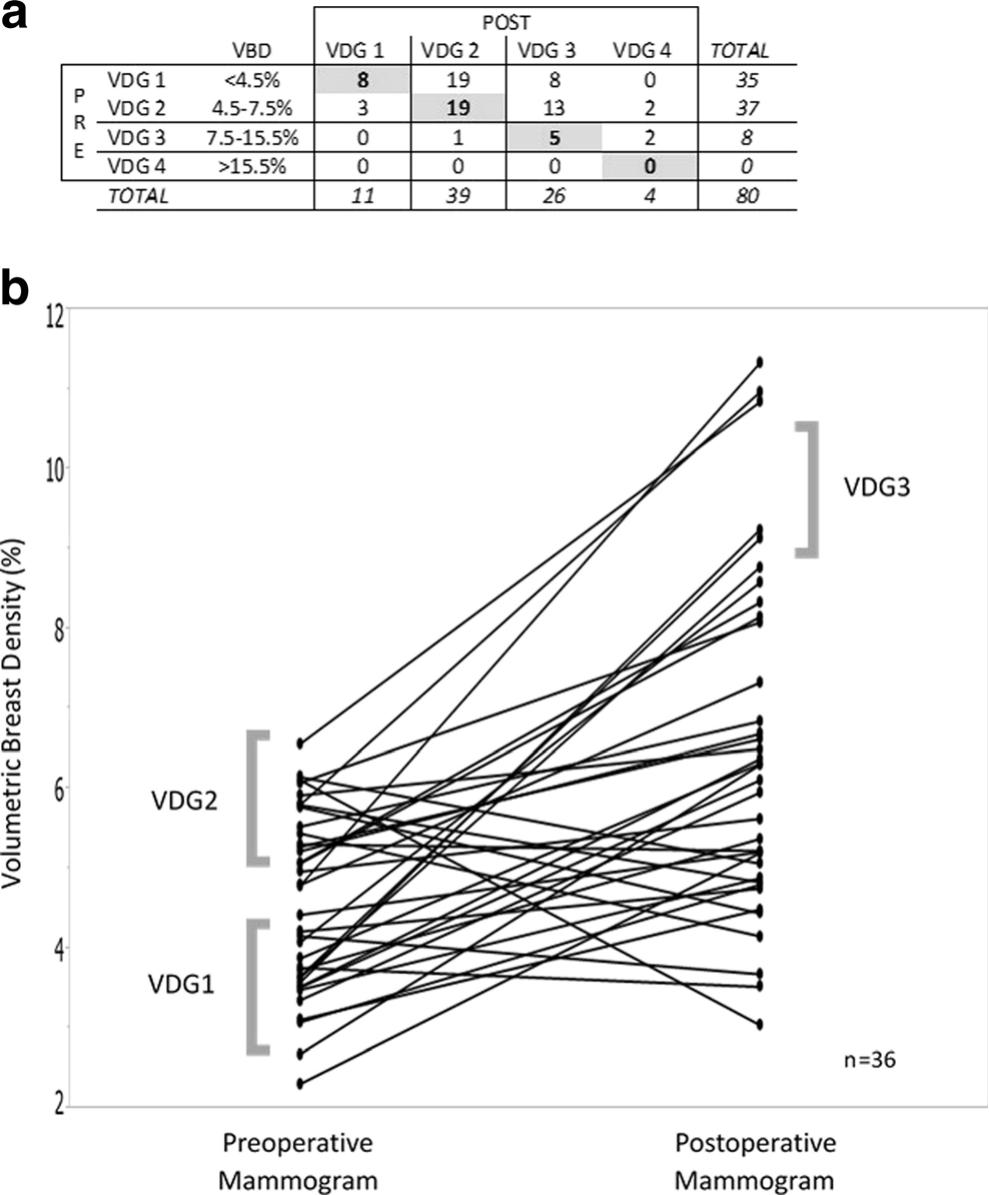
**a** Matrix showing Volpara density grades (VDG) pre and post weight loss surgery. *Shaded bold boxes* indicate the number of patients with unchanged VDG pre and post weight loss surgery. **b** Volumetric breast density (VBD) pre and post weight loss surgery in patients who had an unchanged BI-RADS density score of 2 for the pre and post weight loss surgery mammogram

The distribution of patients into Volpara density grades based on the VBD both pre-WLS and post-WLS is shown in Fig. 4a. The majority of mammograms before WLS were either VDG1 (*n* = 35 or 44 %) or VDG2 (*n* = 37 or 46 %) with none in VDG4, the most dense category (VBD > 15.5 %). However, this profile shifted dramatically to higher VDGs after WLS: 11 VDG1, 39 VDG2, 26 VDG3, and 4 VDG4.

The average VBD of all of the patients with a BI-RADS density of 2 (scattered fibroglandular densities) on their preoperative mammogram was 4.8 %, while the average VBD for all of the patients with a BI-RADS density of 2 on the postoperative mammogram was 7.0 %. Patients with a BI-RADS density of 3 (heterogeneously dense) on the preoperative mammogram had an average VBD of 5.9 %, while patients with a BI-RADS 3 postoperatively had an average VBD of 10.7 %.

To further compare BI-RADS density to VBD, Fig. 4b graphs the Volpara-calculated VBD of the 36 patient subsets who had both a preoperative and postoperative BI-RADS score of 2. The average VBD was 5.16 % on the pre-WLS mammograms and 7.87 % on the post-WLS mammogram with a mean difference of 1.91 ± 2.25 % (*p* < 0.00001, 95 % CI, 1.16, 2.68). As indicated by the brackets, multiple volume density groups (VDGs) are contained within the BI-RADS score of 2. After WLS, the VBD increased to include VDG3.

Average FGV however was lower on the postoperative mammograms compared to the preoperative mammograms for the same BI-RADS score although not statistically significant (data not shown). For a BI-RADS 2 density, the average FGV preoperatively was 67.89 cm^3^ and postoperatively was 56.9cm^3^. For BI-RADS 3 density, the average FGV was 75.01 and 70.68 cm^3^ preoperatively and postoperatively, respectively.

## Discussion

Population studies examining the relationship of BMI and area density measures focus on the obvious contribution of adipose tissue in lowering density but do not take into account how changes in the breast adipose tissue may influence the fibroglandular tissue due to other factors, for example, hormonal changes. With improvements in metabolic regulation after WLS, both physical and biologic changes in the fibroglandular compartment of the breast may also significantly contribute to the reduction in breast cancer risk.

Weight loss surgery has a beneficial effect on obesity, diabetes, hypertension, and the risk for many cancers including postmenopausal breast cancer [43]. The impact of WLS on breast density, itself a significant risk factor for breast cancer, as a potential mechanism for this reduction in breast cancer risk, is unknown. We sought to determine the effect of WLS on VBD and FGV in African American and white obese women. We observed that obese women undergoing WLS had a significant increase in percent VBD following the procedure. This finding does not seem unexpected as adiposity and density are inversely associated with one another [5].

While there are no studies reporting changes in mammographic density in women undergoing WLS, a randomized control trial by Woolcott and colleagues identified no significant change in mammographic density at 1 year in women participating in aerobic exercise versus sedentary women, despite changes in body fat [44]. Our findings are in contrast to this study. This difference in results may be due to the use of a more sensitive measurement tool. Different findings may also be due to the modest change in BMI after exercise versus the drastic drop in BMI after WLS and the likelihood that weight loss through exercise and bariatric surgery may have different effects on breast density. In addition, most subjects in our study were much more obese with an average BMI of 47 (extremely obese, obesity class III) and a postsurgery average BMI of 32 (obesity class I), compared to the average BMI of 29 for the study population in the Woolcott study.

Because studies have reported that both absolute dense area and dense volume are associated with breast cancer risk, we hypothesized that WLS results in a decrease in FGV (dense volume) which may contribute to the decrease in breast cancer risk in this group. We observed a small but significant decrease in FGV following WLS in the entire study population as well as in the subset of postmenopausal women. Both FGV and percent FGV have been described to be more accurate predictors of breast cancer risk than percent dense area [45]. In the study by Shepherd et al., the use of FGV significantly increased risk classification for women with and without breast cancer [45]. Therefore, the decrease in FGV following WLS observed in our study may in fact confer a reduction in breast cancer risk. Failure to observe a decrease in VBD is related to the massive decrease in fat and total breast volume compared to the modest decrease in FGV. VBD in this study is calculated by using Volpara, a software that uses the ratio of total fibroglandular volume and total breast volume thus accounting for the breast thickness. This not only allows examination of the relationship of WLS to density but also provides measures like FGV which reflect the entire dense volume. In a recent report, the variability in repeated measurement of density was evaluated for several automated breast density measurement tools, with Volpara being validated the most reliable making it ideally suited to measure changes in density [30].

While our study does not establish that a reduction in FGV is what imparts the protective effect, it certainly raises the possibility that this may be a potential mechanism that should be investigated further. Adipose tissue is known to produce hormones like estrogen and inflammatory cytokines which may directly affect proliferation of the adjacent epithelial tissue in the breast [46]. A drastic decrease in the adipose tissue may have the potential to cause a change in the FGV as early as a few months after surgery. In fact, in our study population, a decrease in FGV was seen even in patients who had a mammogram only a few months after surgery. Study results from a recently completed prospective clinical trial, examining the effect of weight loss surgery on breast density by using digital mammography and MRI in women who are at increased risk for breast cancer, may provide validation of our findings [47]

Another interesting finding was the lack of change in FGV in patients who remained diabetic after WLS. Although the sample size is too small to make definitive conclusions, one possible explanation for this observation may be related to the proliferative role of the IGF-1 axis on breast epithelium [26, 48]. Our findings thus support future studies to investigate the biochemical changes in the breast tissue before and after WLS.

We report that both premenopausal and postmenopausal women had a decrease in FGV; however, only postmenopausal women experienced a statistically significant decrease in FGV. This difference in the change in FGV pre and post bariatric surgery may reflect the difference in biochemical processes between premenopausal and postmenopausal women, as well as a difference in biological effects of WLS in premenopausal and postmenopausal women. Another possibility is that changes in FGV may have failed to achieve significance because of the small sample size in the premenopausal and perimenopausal group.

There are inherent limitations to this study due its retrospective nature. Although volumetric density measurements by using Volpara are fully automated and do not rely on subjective reader evaluations, they can be affected by compression paddle tilt and mammographic technique, especially with obese individuals where the effect of paddle tilt is greater [49]. In addition, current mammographic breast compression policies do not require pressure standardization which may lead to variation in serial studies [50]. With massive weight loss, the breast thickness in a given patient changes significantly so that this problem may be less of an issue once the patient is leaner but may affect comparisons between presurgery and postsurgery measurements. As with any chart review, information on breast cancer risk factors and other confounding factors was not reliably available on all patients.

The timing of preoperative and postoperative mammogram in relation to the WLS varied; therefore, some patients had their first and only available postoperative mammogram at 60 days, while others did not have one until 3 years. Since the median time to mammogram from surgery was 10 months, it is unlikely that the significant decrease in FGV observed in postmenopausal women in this study is only a result of the normal aging process. It is well known that the majority of weight loss occurs within 6–9 months after bariatric surgery. Because some mammograms were performed before maximal weight loss was achieved, our results likely underestimate the changes measured.

To our knowledge, this is the first published study of its kind to document changes in volumetric breast density and fibroglandular volume following WLS in a unique population of patients. The observations in this study are provocative and warrant a prospective study. Investigations examining the effect of weight loss on breast and stromal tissue may help understand the factors that influence breast cancer risk and provide opportunities for breast cancer prevention.

## Conclusions

Obese women who undergo WLS experience many beneficial health effects, including a reduction in the risk of breast cancer. The mechanism for this is unclear; however, given the various associations between BMI and breast cancer, breast density and breast cancer, and BMI and breast density, we sought to determine how WLS impacts not only breast density but each of the components that comprise density. In this study, through analysis of mammograms by using automated volumetric measurements, we show that fibroglandular volume decreases and volumetric breast density increases following weight loss surgery, with the most significant change observed in postmenopausal women and non-diabetics. Reduction in breast cancer risk following weight loss surgery may thus be due to an effect on fibroglandular volume but not volumetric breast density, and a prospective study to evaluate this hypothesis further is underway.
